# Modulation of Adipose-Derived Mesenchymal Stem/Stromal Cell Transcriptome by G-CSF Stimulation

**DOI:** 10.1155/2020/5045124

**Published:** 2020-02-15

**Authors:** Luz M. Avila-Portillo, Fabio Aristizabal, Angela Riveros, Martin C. Abba, Diego Correa

**Affiliations:** ^1^Departamento de Farmacia, Universidad Nacional de Colombia, Bogotá, Colombia; ^2^Stem Medicina Regenerativa/CryoHoldco, Bogotá, Colombia; ^3^Hospital Militar Central, Bogotá, Colombia; ^4^CINIBA-CIC-PBA, Facultad de Ciencias Médicas, Universidad Nacional de La Plata, La Plata, Argentina; ^5^Department of Orthopaedics-UHealth Sports Medicine Institute, University of Miami, Miller School of Medicine, Miami, FL, USA; ^6^Diabetes Research Institute & Cell Transplant Center, University of Miami, Miller School of Medicine, Miami, FL, USA

## Abstract

Mesenchymal stem/stromal cells (MSCs) exhibit multidifferentiation potential, paralleled with immunomodulatory and trophic properties that make them viable alternative tools for the treatment of degenerative disorders, allograft rejection, autoimmune diseases, and tissue regeneration. MSC functional attributes can be modulated by exposing them to inflammatory-stimulating microenvironments (*i.e.*, priming) before their therapeutic use. Granulocyte-colony stimulating factor (G-CSF) is a cytokine that plays key roles in immune response and hematopoiesis modulation through direct effects on hematopoietic progenitors' proliferation, survival, and mobilization. Despite the established roles of MSCs supporting hematopoiesis, the effects of G-CSF on MSCs biology have not been thoroughly explored. This study reveals that G-CSF has also direct effects on adipose-derived MSCs (ADSCs), modulating their functions. Herein, microarray-based transcriptomic analysis shows that G-CSF stimulation *in vitro* results in modulation of various signaling pathways including ones related with the metabolism of hyaluronan (HA), conferring a profile of cell mobilization to ADSCs, mediated in a cell-intrinsic fashion in part by reducing CD44 expression and HA synthesis-related genes. Collectively, these data suggest a direct modulatory effect of G-CSF on ADSC function, potentially altering their therapeutic capacity and thus the design of future clinical protocols.

## 1. Introduction

Mesenchymal stem/stromal cells (MSCs) have emerged in recent years as potential therapeutic tools for various clinical conditions [1, 2]. This is based on important features such as multidifferentiation potential, a strong capacity to modulate both adaptive and innate immune responses, inducible trophic effects (e.g., angiogenic, mitogenic, antiapoptotic, and antifibrotic), and low immunogenicity that permits allogeneic therapeutic regimes [3, 4]. The adipose tissue constitutes a rich reservoir of MSCs, easily accessible through minimally invasive procedures. Adipose-derived MSCs (ADSCs) exhibit frequencies significantly higher than other sources such as the “standard” bone marrow (BM) [5, 6]. MSCs are starting to be recognized by their ability to sense their surrounding molecular environment [7, 8] and to subsequently react with a broad range of responses involving the paracrine secretion of soluble and microvesicle-packaged molecules including growth factors, cytokines, and miRNAs [9, 10]. Importantly, those responses can be antagonizing, as proinflammatory and anti-inflammatory cascades result from types 1 and 2 MSC phenotypic shifting, respectively, dictated by the specific stimulatory molecular environment [7, 8].

Granulocyte-colony stimulating factor (G-CSF) is a cytokine, whose expression is induced by inflammatory stimuli including IL-1*β*, IL-17, TNF-*α*, and LPS [11]. Its effects are oriented mainly towards increasing the production and mobilization of bone marrow-derived granulocytes of neutrophilic identity as the response [12, 13]. G-CSF exerts its biological effect through binding to its cognate receptor G-CSFR (CD114), which is expressed in several cell types including hematopoietic stem cells (HSPCs), precursor and mature neutrophils, myeloid cell lines, endothelial cells, placenta-derived cells, neurons, ovules, and cardiomyocytes [14]. As for MSCs, Murakami et al. recently demonstrated that ADSCs express CD114 (20.0% ± 1.7), which becomes upregulated to 39.4% ± 4.7 when cells were mobilized *in vivo* with G-CSF [15]. The resulting CD114/G-CSF complex signals through multiple intracellular molecular pathways including Jak-STAT, Ras/MAPK, PI3K, and PKB/AKT cascades, modulating various biological processes including differentiation, migration, and survival [15, 16].

Due to an efficient granulocyte-mobilizing effect, recombinant human G-CSF (rhG-CSF) is now widely used clinically in various conditions such as neutropenia due to chemotherapy and myeloablative therapy followed by HSC transplantation and in HIV infection [17, 18]. More recently, it has been tested as an alternative and/or support to MSCs in studies with ischemic conditions including the myocardium and brain [19, 20] and for degenerative conditions of the central nervous system [21]. Different rhG-CSF formulations are currently approved as a biosimilar of filgrastim (Neupogen) for its clinical use. IOR®leukoCIM is one of them, which has comparable safety and pharmacodynamic and pharmacokinetic profiles demonstrated *in vivo* [22].

Beyond the known effects of G-CSF in HSPCs and its current clinical use, to the best of our knowledge, there is no description regarding potential activities of G-CSF on MSCs and specifically on ADSCs functions. In the present study, we show that two different formulations of rhG-CSF have *in vitro* modulatory effects to the transcriptome of ADSCs, inducing the activation of signaling pathways related to cell proliferation, mobilization, differentiation, and secondary immune responses.

## 2. Materials and Methods

### 2.1. Isolation and Culture of Human Adipose Tissue-Derived MSCs (ADSCs)

The institutional Ethics Committee of the Hospital Militar Central of Bogotá approved this study (2013-049), with all samples (3 independent, de-identified, healthy donors, ages 35, 37, and 39) obtained under written informed consent. Adipose tissues (~40 g) were taken from minimal abdominal lipoaspirate surgery, from which ADSCs were isolated and cultured according to the following protocol. Briefly, adipose tissue was washed with equal volumes of Dulbecco's phosphate-buffered saline (DPBS), and the extracellular matrix (ECM) digested with type I collagenase 0.075% (Invitrogen-Gibco) at 37°C for 30 min. Enzyme activity was then neutralized with low-glucose Dulbecco's modified Eagle's medium (LG-DMEM; Sigma-Aldrich) containing 10% fetal bovine serum (FBS; Invitrogen-Gibco) and 100 U/ml penicillin/streptomycin (PS; Invitrogen-Gibco). Samples were then centrifuged and the cell pellet was seeded in T25 culture flasks in LG-DMEM containing 10% FBS and 100 U/ml PS. The cells were incubated in a humidified atmosphere at 37°C with 5% CO_2_, with the medium changed every 3 to 4 days until the adherent fibroblast-like cells reached 70% confluence. The cells were then passaged twice by trypsin (0.05%) digestion, seeded at a density of 5,000 cells/cm^2^ and finally used as Passage 2. Cell numbers and viability at the time of passage were determined by trypan blue and 7-aminoactinomycin D (7AAD) methods, respectively.

### 2.2. Characterization of MSCs by Immunophenotypic Analysis

Antibodies against the human antigens CD34 (PE), CD45 (FITC), CD105 (PerCP), CD73 (PE), and CD90 (FITC) were obtained from Becton Dickinson. A total of 1 × 10^5^ cells/ml cells were resuspended in 0.2 ml DPBS and incubated with fluorescein isothiocyanate- (FITC-), phycoerythrin- (PE-), or peridinin chlorophyll- (PerCP-) conjugated antibodies for 30 min at room temperature. The fluorescence intensity of the cells was evaluated by flow cytometry (FACS Canto II; Becton Dickinson), and the data were analyzed using the FACS Diva software (Becton Dickinson).

### 2.3. Osteogenic Differentiation Potential of ADSCs

Cells were plated at density of 5 × 10^4^ cells/well in 6-well plates in LG-DMEM containing 10% FBS, allowed to adhere overnight, and replaced with LG-DMEM containing 10% FBS and 1% PS and supplemented with 0.1 mM dexamethasone (Stemcell Technologies), 10 mM b-glycerol phosphate (Stemcell Technologies), and 100 mM ascorbate-2-phosphate (Stemcell Technologies). The medium was changed every 3 days. After 14–21 days, osteoblast differentiation was determined by alkaline phosphatase and von Kossa stainings.

### 2.4. Treatment of ADSCs with rhG-CSF

The optimal dose of both rhG-CSF formulations [Innovator—Neupogen (Roche) and Biosimilar—IOR®leukoCIM (Delta labs)] on ADSCs was evaluated using the 7AAD cell viability staining, analyzed by flow cytometry. ADSCS (1 × 10^5^) from the 3 independent donors were cultured in LG-DMEM containing 20% FBS, 1% PS, and rhG-CSF (100 and 200 ng/ml of either formulation) for 48 hours, with samples taken at 0, 8, 24, and 48 hours for analysis. Based on the resulting data, subsequent experiments were performed with 200 ng/ml and similar evaluation time points. These experiments involved the flow cytometry evaluation (as described above) of CD44 (FITC) to evaluate a migratory phenotype, along with CD90 to attest general MSC phenotypic stability with both rhG-CSF formulations.

For the high-throughput/transcriptomic analysis, 1 × 10^6^ ADSCs were cultured in LG-DMEM containing 20% FBS, 1% PS, and 200 ng/ml of either rhG-CSF formulation for 6 hours. Individual wells were then harvested simultaneously for RNA extraction.

### 2.5. RNA Isolation and Microarray Analysis

Control and stimulated ADSCs were collected by treatment with 0.05% trypsin-EDTA, and total cellular RNA was extracted from pelleted cells and purified using a Quick-RNA™ MiniPrep (Zymo Research) according to the manufacturer's protocol. RNA quality was determined by the OD 260/280 ratio, along with quantification with NanoDrop spectrophotometer (Thermo Scientific).

For microarray gene expression analysis, 2 *μ*g of total RNA from ADSCs treated with either rhG-CSF formulation or labeled with Cy3, while untreated counterparts were labeled with Cy5 (used as reference) for their further hybridization on Agilent SurePrint G3 Human GE v2 8x60K Microarray (Agilent Technologies, Palo Alto, CA, USA), using the RNA derived from the corresponding cells without prior rhG-CSF treatment (labeled with Cy5) as reference. RNA was obtained and processed from the 3 independent donors (biological triplicate), run in duplicate (technical replicas). The hybridization steps were carried out according to the Agilent protocol and images were scanned using a Genepix 4000B microarray scanner (Axon Instruments, Foster City, CA, USA). Image analysis and initial quality control were performed using Agilent Feature Extraction Software v10.2. Raw datasets have been submitted to GEO (GSE139352). We used the Limma Bioconductor package for background adjustment, within and between arrays normalization [23]. To identify significantly up- or downregulated genes within the hybridized samples, we employed the one-class rank products' test (*q* value < 0.05; fold change > 1.5) [24].

### 2.6. Bioinformatic Analysis

Statistical analyses were done with the MultiExperiment Viewer software (MeV 4.9) [25]. The number and identity of genes commonly affected in both models were determined with Venn mapping. Functional enrichment analyses were performed with InnateDB (https://www.innatedb.com/index.jsp), REVIGO (http://revigo.irb.hr/), and Cytoscape software (https://cytoscape.org).

## 3. Results

### 3.1. Isolation and Characterization of ADSCs

**Figure 1:**
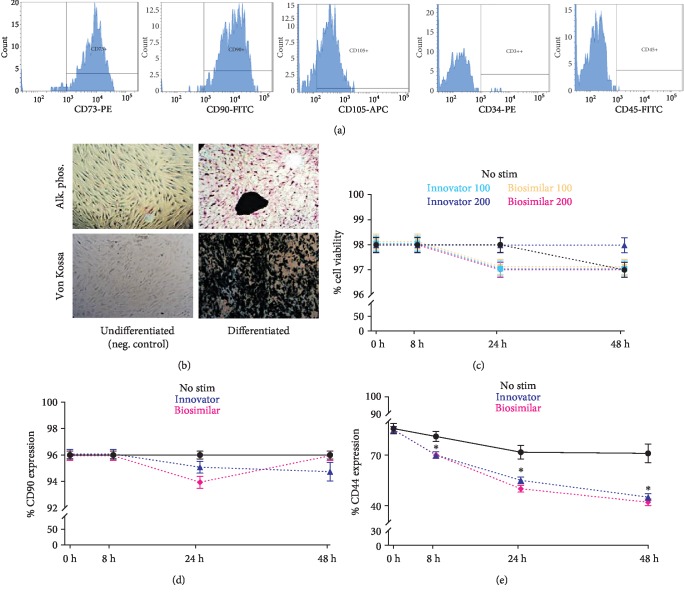
Characterization of ADSC and stimulation with rhG-CSF. (a) ADSC immunophenotypic assessment as positive for CD73, CD90, and CD105 and negative for CD34 and CD45. (b) Osteogenic differentiation of ADSCs after 14–21 days of induction, assessed by alkaline phosphatase (Alk. Phos.) and von Kossa stainings, showing adequate mineralization of the ECM. (c) Cell viability assessed through 7AAD assay analyzed at 8, 24, and 48 h, showing no toxicity with either formulation at both doses. (d, e) ADSC stimulation with both formulations at 200 ng/ml, showing CD90 expression unaltered during the same time period (d), while CD44 was significantly reduced as early as 8 hours post stimulation. ^∗^*p* < 0.001 when compared to time 0 for each condition.

Processed adipose tissue yielded ADSCs in culture with fibroblast-like appearance and exhibiting fibroblast colony-forming units (CFU-F) typical of MSC. Flow cytometric analysis showed that ADSCs derived from all donors were positive for the typical MSC markers such as CD73, CD90, and CD105, while negative for the hematopoietic markers CD34 and CD45 (Figure 1(a)). Expanded ADSCs exhibited a conserved potential to differentiate into osteoblasts (Figure 1(b)), indicating that all populations were comprised of multipotent MSC.

### 3.2. Effect of G-CSF on ADSC Viability and Phenotype

The 7AAD viability assay showed that both formulations of rhG-CSF (at both dose regimes) had no toxic effects on ADSCs up to 48 hours, evidencing cell survival between 97% and 98% throughout the stimulatory period (Figure 1(c)). Based on this information, we used 200 ng/ml for subsequent experiments. Importantly, CD90 was not impacted by G-CSF treatment, which maintained expression between 94% and 96% (Figure 1(d)), thus evidencing a phenotypic stabilization of ADSCs during the stimulation.

### 3.3. Effect of G-CSF on Expression of CD44 by ADSCs

Unlike CD90, flow cytometry analysis revealed that CD44 presence on ADSC was significantly reduced by rhG-CSF stimulation throughout the treatment period, and as early as 8 hours. CD44 experienced a reduction from ~85% (baseline) to ~45% with rhG-CSF (*p* < 0.05), while control cells had a minor, yet not significant reduction to ~75% (Figure 1(e)).

### 3.4. Effect of G-CSF on ADSC Gene Expression Profiles *In Vitro* and Common Bioprocesses

The primary goal of this study was to identify commonly modulated pathways among biosimilar and innovator G-CSF in ADSC. Microarray-based gene expression profiling was performed on cell samples isolated from the same individuals (*n* = 3), after 6 hours of treatment with biosimilar or innovator G-CSFs and their corresponding untreated counterpart samples used as controls. First, we performed an evaluation of gene expression profiles associated with biosimilar and innovator G-CSF treatments, to identify the most representative differentially expressed transcripts for each drug. Second, we compared the biosimilar and innovator G-CSF gene expression signatures, to identify the commonly modulated transcripts by both drugs. Third, functional enrichment analysis was performed to detect the G-CSF-modulated pathways in ADSC.

**Figure 2:**
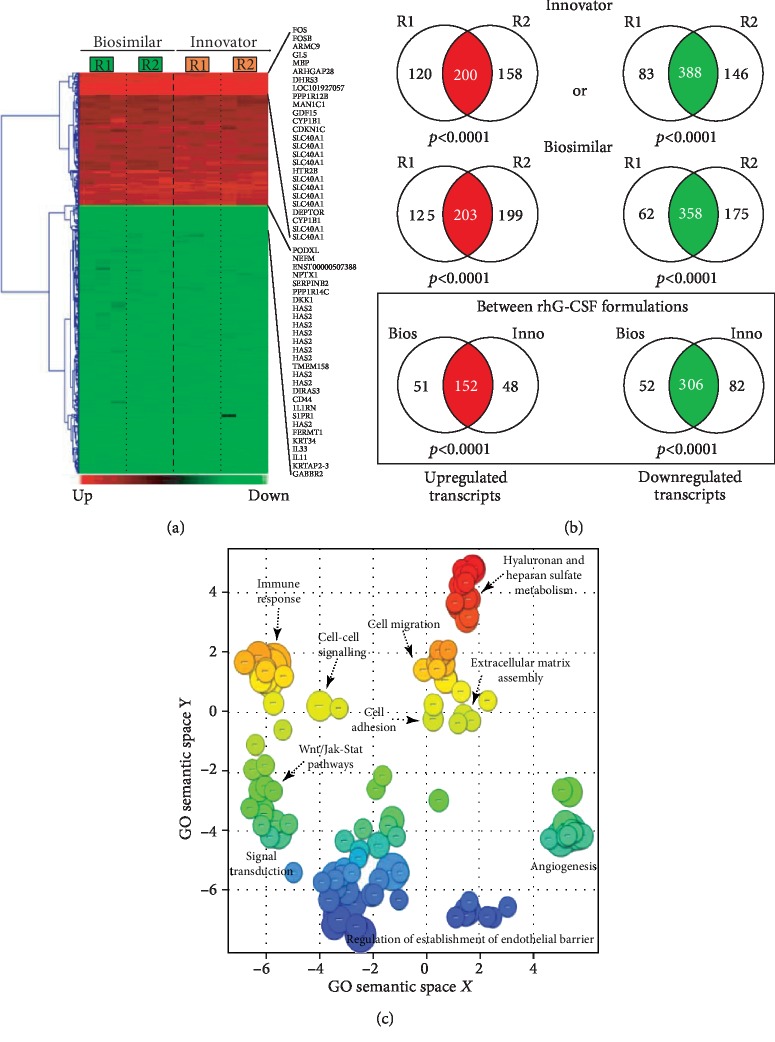
Hierarchical cluster and Gene Ontology analysis of differentially expressed genes in ADSCs treated with G-CSF. (a) Heat map of differentially expressed genes involved in G-CSF responses in ADSCs. (b) Venn diagrams showing high consistency between the two technical replicas (R1 and R2) run for each biological triplicate for both Innovator and Biosimilar formulations (top 2 rows), and between them in terms of upregulated and downregulated genes (lower row). (c) Functional annotation showed that genes could be grouped into a limited number of biological categories. Most of the genes upregulated in ADSCs were enriched in innate immune response, differentiation, and MAPK cascade (*p* value < 0.001), while most of the genes downregulated are involved in cell migration and cell adhesion (*p* value < 0.001).

This statistical analysis revealed 458 commonly differentially expressed transcripts across biosimilar and innovator G-CSF (fold change ≥ 1.5; *q* < 0.05). Among the 458 transcripts, 152 were upmodulated and 306 were downmodulated transcripts in G-CSF treatments (Figures 2(a) and 2(b)). The most commonly overexpressed transcripts in G-CSF treatments were *CDKN1C*, *CYP1B1*, *SLC40A1*, *HTR2B*, and *DEPTOR*, and the most commonly downexpressed were *NEFM*, *PODXL*, *KRT34*, *IL33*, and *KRTAP2-3*. Functional enrichment analysis of commonly deregulated transcripts revealed bioprocess associated with innate immune response, differentiation, and PI3K and MAPK signaling pathways (*p* value < 0.001) as well as cell migration and cell adhesion (*p* value < 0.001) (Figure 2(c) and Supplementary Tables [Supplementary-material supplementary-material-1]).

**Figure 3:**
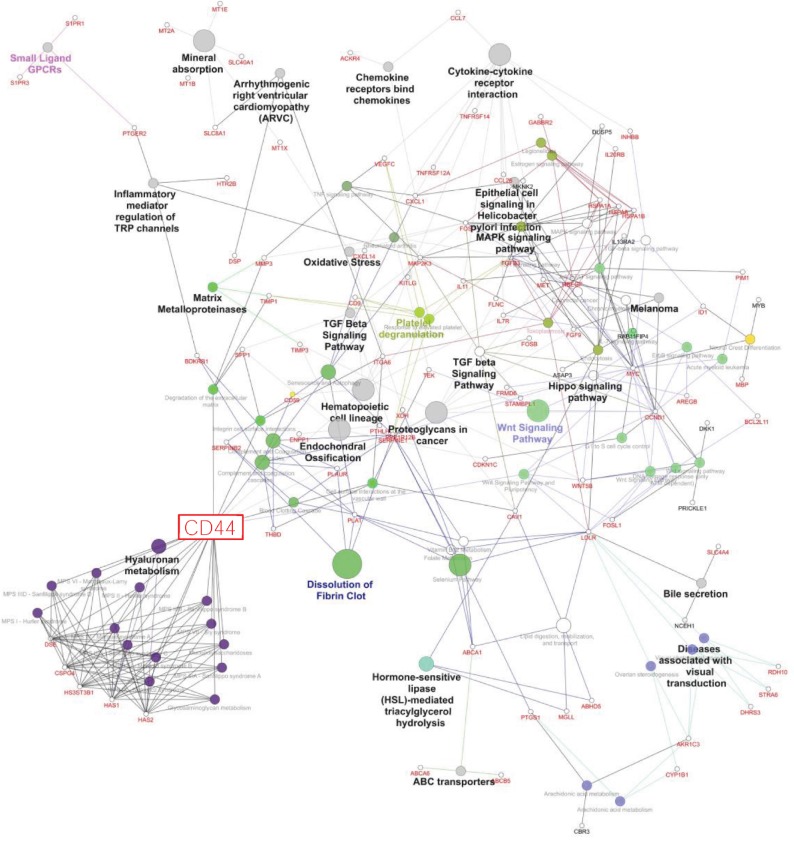
Molecular interaction networks and integration with gene expression profiles in ADSCs exposed to G-CSF *in vitro* (Cytoscape).

The commonly modulated bioprocess associated with the rhG-CSF treatment of ADSCs can be grouped into two groups: (i) upstream events of G-CSF treatment including crosstalk among MAPK signaling, JAK-STAT and Wnt signaling, and Hippo pathway, which are related to proliferation and cell differentiation and (ii) downstream effects including regulation of the actin cytoskeleton and cell mobilization (Figure 3).

We selected genes for transcription factors playing a key role in ADSC cell cycle regulation: HDAC2 and CCND1 (*p* = 0.03) and JAK-STAT pathway CCND1 and STAT1 (*p* = 0.04). These transcription factors were found significantly upregulated in rhG-CSF-treated cells. This finding might be due to a direct transcriptional activation by the drugs on ADSCs.

## 4. Discussion

The results for this study, the first work describing the effect of in vitro stimulation for 6 hours of G-CSF on the ADSC transcriptome, demonstrate that both G-CSFs (the innovative and biosimilar) modulate the same gene expression signature in ADSC cells. An interesting fact is given that the development and manufacturing of biosimilar drugs are increasing and there is a great interest in the continuous development of analytical methods that can be used to efficiently and effectively characterize biosimilars. Transcriptomic analysis involves the study of gene expression through the levels of mRNA present in cells exposed to treatment in relation to a control test, which provides insight into which biological pathways are active or inactive within the cell. Therefore, the analysis of gene expression could be a tool to evaluate the bioequivalence of biosimilar drugs.

Bioinformatic and data mining analysis suggest that G-SCF induces upmodulation and downmodulation of genes involved in several signaling pathways. Functional analysis suggests enrichments of signaling pathways related with cellular metabolism, activation of TLRs (innate immunity), induction of the PI3K-Akt signaling pathway and activation of the NFkB and MAP pathway (innate immunity), and downregulation of genes associated with processes of cell migration, ECM remodeling, and hematopoietic lineage. These data are important because they open the possibility of deepening the relationship of this drug and influenced ADSC cells when a patient undergoes therapeutic protocols of stimulation with G-CSF.

The discussion of our results will be centered on the possibility that ADSCs following G-CSF stimulation may migrate, as their hematopoietic stem cells (HSPCs) counterpart. This is a result of the regulation and interactions between the pleiotropic signaling pathways and specific cellular regulators identified in the transcriptome.

CD44 is a molecule expressed by many immune cells and progenitors (i.e., hematopoietic stem/progenitor cells—HSPCs). It is involved in several biological processes exhibiting dual roles in cell proliferation, adhesion, and signaling molecules [26]. CD44 has a documented activity inducing the mobilization and migration of immune cells and progenitors from and to the bone marrow [27] and to inflammatory sites and lymph nodes [28, 29], maintaining them circulating during inflammation [30]. While CD44 expression in ADSCs has been documented [31], bone marrow-derived MSC has been only shown to be induced by inflammation and G-CSF [32].

We are showing by both transcriptomic and flow cytometric analysis post stimulations with G-SCF a significant reduction in CD44, a biologically interesting result given its interaction with multiple ligands including hyaluronate (HA), a highly expressed molecule in mesenchymal cells of different origins [33]. CD44 activity has been well established in HSPC regulation [34]. CD44/HA binding promotes cell adhesion via cytokines/chemokines, quiescence, and resistance to hypoxia in HSPCs [35]. Studies with mice have shown that in HSPCs, the use of anti-CD44 antibodies is associated with alteration in their “homing” to BM and spleen [36]. In contrast, studies in CD44 KO mice (knockout) showed no defects in this process [37]; however, a reduction was found in the output of BM myeloid progenitor cells to the circulation [27]. In humans, it has been reported that administration of G-CSF appears to result in faster mobilization of CD34+ HSPCs [38, 39]. Furthermore, this mobilization is associated with a downregulation in both CD44 and CD31 from days 3 to 5 of continuous intravenous administration, measured in peripheral blood CD34+ [40].

Based on the established mechanism involving the support of CD44 reduction in the mobilization of HSPCs, we hypothesized that it could confer a similar migratory role to ADSCs after stimulation with G-CSF. In fact, the transcriptomic data supports that hypothesis. Our results showed a concomitant reduced expression of *HAS1* and *HAS2*, responsible genes for synthesizing hyaluronan (HA) at the inner face of the plasma membrane [41]. Therefore, a probable synergistic effect by the reduced CD44, *HAS1* and *HAS2* could lead to a reduction in the production and/or activity of HA, negatively impacting its activity as an ECM ligand anchoring ADSCs.

## 5. Conclusions

**Figure 4:**
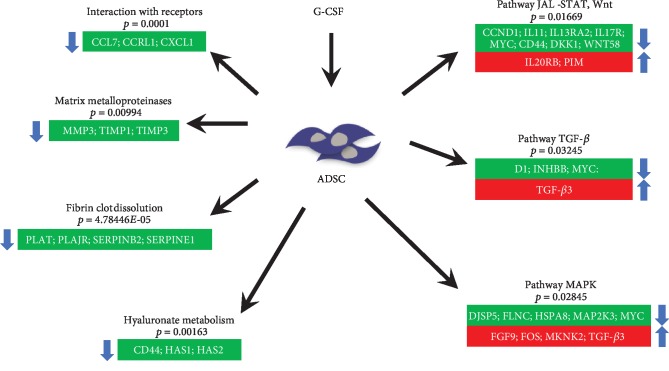
Most relevant signaling pathways and genes altered (upregulated in red and downregulated in green) in ADSCs after stimulation with G-CSF.

In conclusion, the G-CSF impacts the transcriptome of the ADSCs modulating various pathways including ones related with the metabolism of hyaluronan, conferring a profile of cell mobilization (Figure 4). Collectively, to the best of our knowledge, this is the first time that ADSC migratory capacity is linked with G-CSF stimulation and specifically through a reduction in CD44. This could potentially alter ADSC therapeutic capacity and thus the design of future clinical protocols, for instance, by stimulating/licensing them with G-CSF prior to administration.

## Data Availability

The raw datasets used to support the findings of this study have been deposited in the Gene Expression Omnibus (GEO) repository with the identifier GSE139352.

## Additional Points

*Highlights*. Innovator and biosimilar G-CSF induce comparable transcriptomic responses in ADSCs. G-CSF favors a cell migration response mainly by altering hyaluronan (HA) metabolism pathways. G-CSF downregulates CD44 and HAS1/HAS2 genes. G-CSF decreases CD44 expression in ADSCs in culture over time.

## Supplementary Materials

Supplementary Materials Two tables are presented with significantly enriched molecular pathways and upregulated (Supplementary Table 1) and downregulated (Supplementary Table 2) genes in ADSCs in vitro after stimulation with G-CSF.

## References

[1] Wang Y., Chen X., Cao W., Shi Y. (2014). Plasticity of mesenchymal stem cells in immunomodulation: pathological and therapeutic implications. *Nature Immunology*.

[2] Phinney D. G., Galipeau J. (2019). Manufacturing mesenchymal stromal cells for clinical applications: A survey of Good Manufacturing Practices at U.S. academic centers. *Cytotherapy*.

[3] Caplan A. I., Correa D. (2011). The MSC: an injury drugstore. *Cell Stem Cell*.

[4] Ankrum J. A., Ong J. F., Karp J. M. (2014). Mesenchymal stem cells: immune evasive, not immune privileged. *Nature Biotechnology*.

[5] Zuk P. A., Zhu M., Mizuno H. (2001). Multilineage cells from human adipose tissue: implications for cell-based therapies. *Tissue Engineering*.

[6] Zuk P. A., Zhu M., Ashjian P. (2002). Human adipose tissue is a source of multipotent stem cells. *Molecular Biology of the Cell*.

[7] Waterman R. S., Tomchuck S. L., Henkle S. L., Betancourt A. M. (2010). A new mesenchymal stem cell (MSC) paradigm: polarization into a pro-inflammatory MSC1 or an immunosuppressive MSC2 phenotype. *PLoS One*.

[8] Bernardo M. E., Fibbe W. E. (2013). Mesenchymal stromal cells: sensors and switchers of inflammation. *Cell Stem Cell*.

[9] Willis G. R., Kourembanas S., Mitsialis S. A. (2017). Toward exosome-based therapeutics: isolation, heterogeneity, and fit-for-purpose potency. *Frontiers in Cardiovascular Medicine*.

[10] Cheng L., Zhang K., Wu S., Cui M., Xu T. (2017). Focus on mesenchymal stem cell-derived exosomes: opportunities and challenges in cell-free therapy. *Stem Cells International*.

[11] Hareng L., Hartung T. (2002). Induction and regulation of endogenous granulocyte colony-stimulating factor formation. *Biological Chemistry*.

[12] Manz M. G., Miyamoto T., Akashi K., Weissman I. L. (2002). Prospective isolation of human clonogenic common myeloid progenitors. *Proceedings of the National Academy of Sciences of the United States of America*.

[13] Bussolino F., Wang J. M., Defilippi P. (1989). Granulocyte- and granulocyte-macrophage-colony stimulating factors induce human endothelial cells to migrate and proliferate. *Nature*.

[14] Larsen A., Davis T., Curtis B. M. (1990). Expression cloning of a human granulocyte colony-stimulating factor receptor: a structural mosaic of hematopoietin receptor, immunoglobulin, and fibronectin domains. *The Journal of Experimental Medicine*.

[15] Murakami M., Hayashi Y., Iohara K., Osako Y., Hirose Y., Nakashima M. (2015). Trophic effects and regenerative potential of mobilized mesenchymal stem cells from bone marrow and adipose tissue as alternative cell sources for pulp/dentin regeneration. *Cell Transplantation*.

[16] Yoshikawa A., Murakami H., Nagata S. (1995). Distinct signal transduction through the tyrosine-containing domains of the granulocyte colony-stimulating factor receptor. *The EMBO Journal*.

[17] Welte K. (2014). G-CSF: filgrastim, lenograstim and biosimilars. *Expert Opinion on Biological Therapy*.

[18] Zanet E., Michieli M., Tirelli U. (2016). Autologous stem cell transplantation in HIV-positive patients affected by relapsed/partially responding lymphoma: let it be. *Expert Review of Hematology*.

[19] Peña B., Laughter M., Jett S. (2018). Injectable hydrogels for cardiac tissue engineering. *Macromolecular Bioscience*.

[20] Peña I. D., Borlongan C. V. (2015). Translating G-CSF as an adjunct therapy to stem cell transplantation for stroke. *Translational Stroke Research*.

[21] Wu J. H., Qiao P. F., Sun X. Y. (2019). Evaluation and application of donors with primary central nervous system tumors. *Clinical Transplantation*.

[22] Licollari A., Riddle K., Taylor S. R., Ledon N., Bolger G. T. (2017). Safety and biosimilarity of ior&reg; LeukoCIM compared to Neupogen&reg; based on toxicity, pharmacodynamic, and pharmacokinetic studies in the Sprague-Dawley rat. *Journal of Pharmaceutical Sciences*.

[23] Breitling R., Armengaud P., Amtmann A., Herzyk P. (2004). Rank products: a simple, yet powerful, new method to detect differentially regulated genes in replicated microarray experiments. *FEBS Letters*.

[24] Saeed A. I., Sharov V., White J. (2003). TM4: a free, open-source system for microarray data management and analysis. *BioTechniques*.

[25] Smid M., Dorssers L. C. J., Jenster G. (2003). Venn mapping: clustering of heterologous microarray data based on the number of co-occurring differentially expressed genes. *Bioinformatics*.

[26] Kothapalli D., Flowers J., Xu T., Puré E., Assoian R. K. (2008). Differential activation of ERK and Rac mediates the proliferative and anti-proliferative effects of hyaluronan and CD44. *The Journal of Biological Chemistry*.

[27] Avigdor A., Goichberg P., Shivtiel S. (2004). CD44 and hyaluronic acid cooperate with SDF-1 in the trafficking of human CD34+ stem/progenitor cells to bone marrow. *Blood*.

[28] Baaten B. J. G., Li C.-R., Bradley L. M. (2010). Multifaceted regulation of T cells by CD44. *Communicative & Integrative Biology*.

[29] Nácher M., Blázquez A. B., Shao B. (2011). Physiological contribution of CD44 as a ligand for E-selectin during inflammatory T-cell recruitment. *The American Journal of Pathology*.

[30] Xu H., Manivannan A., Crane I., Dawson R., Liversidge J. (2008). Critical but divergent roles for CD62L and CD44 in directing blood monocyte trafficking in vivo during inflammation. *Blood*.

[31] Mildmay-White A., Khan W. (2017). Cell surface markers on adipose-derived stem cells: a systematic review. *Current Stem Cell Research & Therapy*.

[32] Qu B., Chu Y., Zhu F. (2017). Granulocyte colony-stimulating factor enhances the therapeutic efficacy of bone marrow mesenchymal stem cell transplantation in rats with experimental acute pancreatitis. *Oncotarget*.

[33] Bourguignon L. Y. W., Peyrollier K., Gilad E., Brightman A. (2007). Hyaluronan-CD44 interaction with neural Wiskott-Aldrich syndrome protein (N-WASP) promotes actin polymerization and ErbB2 activation leading to beta-catenin nuclear translocation, transcriptional up-regulation, and cell migration in ovarian tumor cells. *The Journal of Biological Chemistry*.

[34] Cao H., Heazlewood S. Y., Williams B. (2015). The role of CD44 in fetal and adult hematopoietic stem cell regulation. *Haematologica*.

[35] Misra S., Heldin P., Hascall V. C. (2011). Hyaluronan-CD44 interactions as potential targets for cancer therapy. *FEBS Journal*.

[36] Lemoli R. M., Catani L., Talarico S. (2006). Mobilization of bone marrow-derived hematopoietic and endothelial stem cells after orthotopic liver transplantation and liver resection. *Stem Cells*.

[37] Protin C., Abravanel F., Alric L. (2019). Ribavirin for Chronic Hepatitis E Virus Infection in Ibrutinib-Exposed Patients. *Open Forum Infectious Diseases*.

[38] Philpott N. J., Prue R. L., Marsh J. C. W., Gordon‐Smith E. C., Gibson F. M. (2003). G-CSF-mobilized CD34+peripheral blood stem cells are significantly less apoptotic than unstimulated peripheral blood CD34+cells: role of G-CSF as survival factor. *British Journal of Haematology*.

[39] Machaczka M., Hägglund H., Staver E. (2017). G-CSF mobilized peripheral blood stem cell collection for allogeneic transplantation in healthy donors: analysis of factors affecting yield. *Journal of Clinical Apheresis*.

[40] Lee S., Im S. A., Yoo E. S. (2000). Mobilization kinetics of CD34(+) cells in association with modulation of CD44 and CD31 expression during continuous intravenous administration of G-CSF in normal donors. *Stem Cells*.

[41] Baggenstoss B. A., Harris E. N., Washburn J. L., Medina A. P., Nguyen L., Weigel P. H. (2017). Hyaluronan synthase control of synthesis rate and hyaluronan product size are independent functions differentially affected by mutations in a conserved tandem B-X7-B motif. *Glycobiology*.

