# Supplementary material for: Modulation of Adipose-Derived Mesenchymal Stem/Stromal Cell Transcriptome by G-CSF Stimulation
Source: Stem Cells Int. 2020 Feb 15;2020:5045124. doi: 10.1155/2020/5045124 (PMC7044478; doi:10.1155/2020/5045124)
Supplement: Supplementary Materials — Two tables are presented with significantly enriched molecular pathways and upregulated (Supplementary Table 1) and downregulated (Supplementary Table 2) genes in ADSCs in vitro after stimulation with G-CSF. [file 5045124.f1.pdf]

Supplementary Materials

Two tables are presented with Significantly Enriched Molecular Pathways and up-regulated (Supplementary Table 1) and down-regulated (Supplementary Table 2) genes in ADSCs in *vitro* after stimulation with G-CSF.

Cascade	P-value	Genes
**Signaling by Retinoic Acid**	0,00093	RDH10; AKR1C3; DHRS3
**BH3-only proteins associate with and inactivate anti-apoptotic BCL-2 members**	0,00080	BCL2L11; BMF
**Lipoprotein metabolism**	0,01047	ABCA1; LDLR
**Arachidonic acid metabolism**	0,02345	AKR1C3; CYP1B1
**Retinoid metabolism and transport**	0,01539	AKR1C3; LDLR
**Activation of BH3-only proteins**	0,00783	BCL2L11; BMF
**Metabolism of fat-soluble vitamins**	0,02189	AKR1C3; LDLR
**Intrinsic Pathway for Apoptosis**	0,01540	BCL2L11; BMF
**Co-stimulation by the CD28 family**	0,04054	TNFRSF14; TRIB3
**ABC-family proteins mediated transport**	0,01745	ABCA6; ABCB5
**PI3K Cascade**	0,04772	FGF9; TRIB3
**Lipid digestion, mobilization, and transport**	0,03955	ABCA1; LDLR
**MyD88:Mal cascade initiated on plasma membrane**	0,03218	PELI2; FOS

Supplementary Table 1: Significantly Enriched Molecular Pathways and up-regulated genes in ADSCs in *vitro* after stimulation with G-CSF.

Cascade	p- value	Genes
**Mineral absorption**	0.00011	*MT2A; MT1A; MT1X; MT1B; SLC8A1; MT1E*
**Complement and coagulation cascades**	0.00015	*THBD; SERPINB2; SERPINE1; PLAUR; BDKRB1; PLAT; CD59*
**Hematopoietic cell lineage**	0.00029	*IL11; KITLG; CD9; ITGA6; CD59; IL7R; CD44*
**Cytokine-cytokine receptor interaction**	0.00126	*IL11; KITLG; TNFRSF12A; CCL7; VEGFC; INHBB; CXCL1; IL7R; CXCL14; MET; CCL26*
**Hippo signaling pathway**	0.00606	*FRMD6; WNT5B; CCND1; MYC; SERPINE1; ID1; AREG*
**PI3K-Akt signaling pathway_**	0.02089	*KITLG; CCND1; MYC; MYB; SPP1; VEGFC; ITGA6; TEK; IL7R; MET*
**Focal adhesión**	0.02372	*CCND1; CAV1; SPP1; VEGFC; ITGA6; FLNC; MET*
**Regulation of lipolysis in adipocytes**	0.04626	*ABHD5; MGLL; PTGS1*
**Glycosaminoglycan biosynthesis - heparan sulfate / heparin**	0.04846	*HS3ST3B1; XYLT1*
**Wnt signaling pathway**	0.05010	*FOSL1; WNT5B; CCND1; MYC; DKK1*
**Cell adhesion molecules (CAMs)**	0.05010	*CLDN4; CLDN14; NRXN3; LRRC4; ITGA6*

Supplementary Table 2: Significantly Enriched Molecular Pathways and down-regulated genes in ADSCs in *vitro* after stimulation with G-CSF.
